# Spatiotemporal reproduction number with Bayesian model selection for evaluation of emerging infectious disease transmissibility: an application to COVID-19 national surveillance data

**DOI:** 10.1186/s12874-023-01870-3

**Published:** 2023-03-14

**Authors:** Chawarat Rotejanaprasert, Andrew B. Lawson, Richard J. Maude

**Affiliations:** 1 Department of Tropical Hygiene, Faculty of Tropical Medicine, Mahidol University, Bangkok, Thailand; 2 Mahidol-Oxford Tropical Medicine Research Unit, Faculty of Tropical Medicine, Mahidol University, Bangkok, Thailand; 3 Department of Public Health Sciences, Medical University of South Carolina, Charleston, SC USA; 4 Usher Institute, University of Edinburgh, Edinburgh, UK; 5 Harvard T.H. Chan School of Public Health, Harvard University, Cambridge, MA USA; 6 Centre for Tropical Medicine and Global Health, Nuffield Department of Medicine, University of Oxford, Oxford, UK; 7 The Open University, Milton Keynes, UK

**Keywords:** Spatiotemporal, Reproduction number, Surveillance, Covid19, Thailand

## Abstract

**Background:**

To control emerging diseases, governments often have to make decisions based on limited evidence. The effective or temporal reproductive number is used to estimate the expected number of new cases caused by an infectious person in a partially susceptible population. While the temporal dynamic is captured in the temporal reproduction number, the dominant approach is currently based on modeling that implicitly treats people within a population as geographically well mixed.

**Methods:**

In this study we aimed to develop a generic and robust methodology for estimating spatiotemporal dynamic measures that can be instantaneously computed for each location and time within a Bayesian model selection and averaging framework. A simulation study was conducted to demonstrate robustness of the method. A case study was provided of a real-world application to COVID-19 national surveillance data in Thailand.

**Results:**

Overall, the proposed method allowed for estimation of different scenarios of reproduction numbers in the simulation study. The model selection chose the true serial interval when included in our study whereas model averaging yielded the weighted outcome which could be less accurate than model selection. In the case study of COVID-19 in Thailand, the best model based on model selection and averaging criteria had a similar trend to real data and was consistent with previously published findings in the country.

**Conclusions:**

The method yielded robust estimation in several simulated scenarios of force of transmission with computing flexibility and practical benefits. Thus, this development can be suitable and practically useful for surveillance applications especially for newly emerging diseases. As new outbreak waves continue to develop and the risk changes on both local and global scales, our work can facilitate policymaking for timely disease control.

**Supplementary Information:**

The online version contains supplementary material available at 10.1186/s12874-023-01870-3.

## Background

**Fig. 1:**
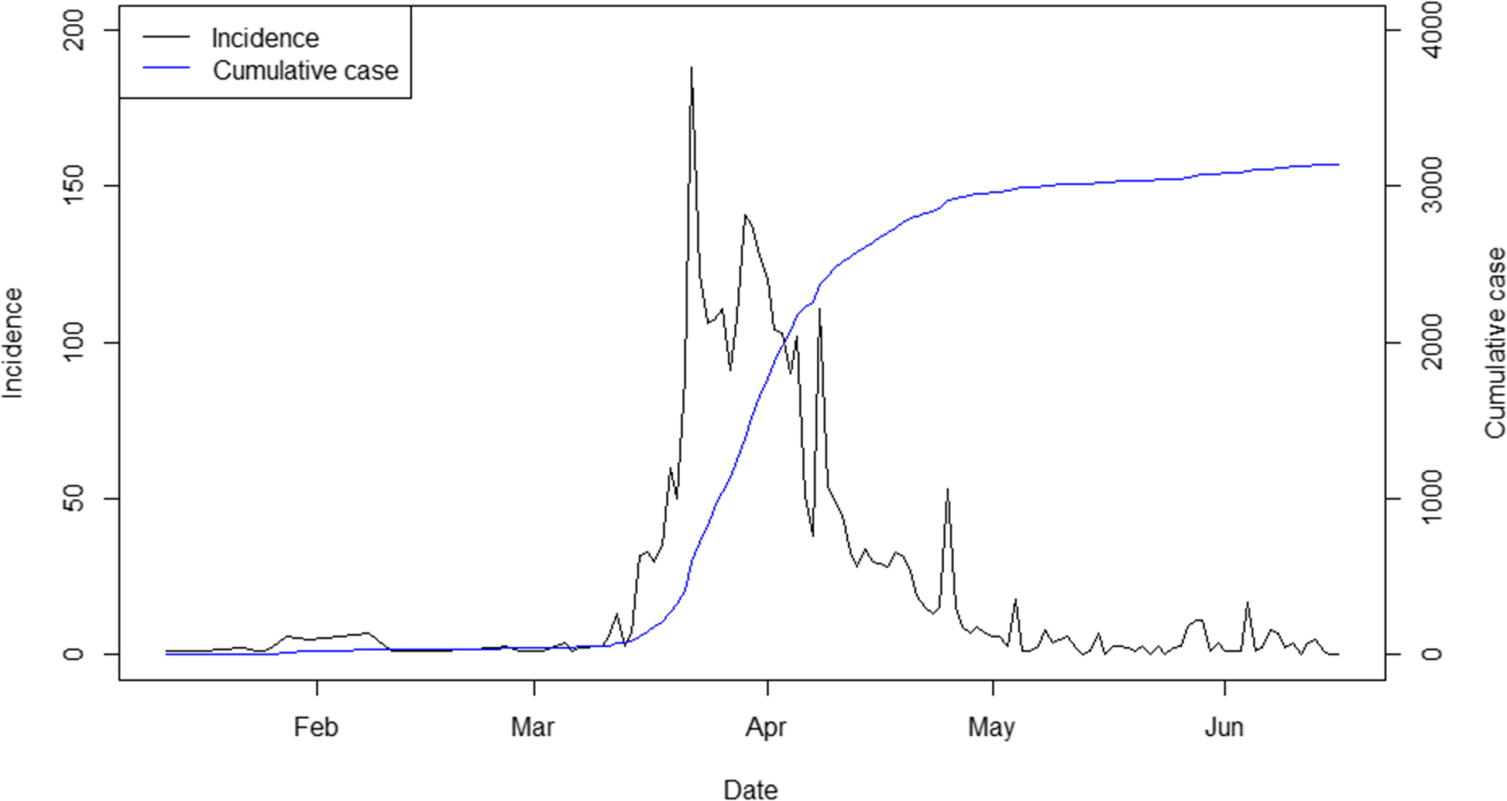
New and cumulative COVID-19 cases in Thailand from January to June 2020

Since the emergence of the new severe acute respiratory syndrome coronavirus (SARS-CoV) at the end of 2019, it has spread rapidly around the world, infecting millions of people. By early 2020, COVID-19 outbreaks had appeared in many countries with one of the first affected being Thailand. Figure 1 shows the numbers of new and cumulative cases in Thailand during the first outbreak in 2020. Due to the absence of an effective treatment or vaccine through much of 2020, strategies to counter the epidemic focused on physical distancing, mask wearing, hand hygiene and restricted international and local travel to slow transmission and avoid overwhelming of the health system. Following introduction of vaccines, there have been challenges of limited availability and limited efficacy against transmission, particularly of more recent variants. Thus, transmission prevention through other measures has continued to be employed. Both the planning and public acceptance of such measures have been highly dependent upon the use of epidemiological models to probe the potential impact of interventions. The effectiveness of, and decision-making for, those interventions needs to be continuously monitored and evaluated.

The decision-making problem faced by government policymakers during a pandemic crisis like COVID-19 is not trivial. In this circumstance governments often have to make decisions based on very uncertain information. Since the disease situation can be very dynamic, it is particularly important to have updated information for prompt decision making. Various transmissibility metrics can be adopted to inform the planning of control measures depending on the available data. The reproduction number is a key threshold widely used to assess the transmission dynamics of an emerging infection including COVID-19 [1, 2]. The basic form of reproduction number (*R*_*0*_) yields the average number of secondary cases generated per case in a fully susceptible population. Although the basic reproduction number may be valuable to understand the pattern of disease, it assumes that the outbreak first occurs in a population with full susceptibility, and hence this quantity is essentially a theoretically defined number and may be less useful to monitor and evaluate the dynamics of disease transmission in real populations [3–5]. An wide range of methods have been proposed to estimate the basic reproduction number (see examples [6, 7]).

The effective or temporal reproductive number, denoted as *R*_e_ or *R*_*t*_, is used to estimate the expected number of new cases caused by an infectious person in a partially susceptible population [8, 9]. If the reproduction number is less than one, the disease occurs in isolated clusters as self-limited chains of transmission, whilst a reproduction number larger than or equal to one indicates sustained transmission. Estimation of* R*_*t*_ has been used to assess how changes in public health policies and interventions have affected transmission at specific points in time, including for COVID-19 in many countries (see examples [10–12]). While the temporal dynamic is captured by *R*_*t*_, the dominant approach is currently based on modeling that implicitly treats people within a population as geographically well mixed. Although mathematical modeling could potentially be used to calculate the spatially varying *R*_*t*_, this would require detailed information which could be challenging as there would be limited evidence with emerging diseases. While some such methods include differential contact by demographic and age-specific groups, those models presently in wide use do not incorporate spatial heterogeneity at local scales [13]. Previous studies however have presented evidence of heterogeneity in social relationships at regional, urban, and suburban scales [14, 15], with these variations in disease spread [3, 16], neighborhood identification, and development [17]. If each individual is not socially well mixed at local scales, it is then probable that diffusion of infected cases via interpersonal contacts will likely deviate from the assumption of uniformly mixed characteristics. To incorporate spatial heterogeneity, this estimation challenge can be further extended to, and addressed within, a small area modeling framework which can produce sensible estimates by sharing information between neighboring areas.

Another aspect that needs to be considered is the model specification for reproduction number estimation. A key ingredient to compute reproduction numbers is the generation interval [8, 18] and misspecification of this component can create large potential bias in transmission estimates which in turn can mislead the public health response. Selecting an appropriate generation time is one of the most important aspects of the calculation, and this can become very challenging when space–time structures are present in the data. Many methods, such as variable selection, transformation selection, model selection, and model averaging, have been proposed and explored to achieve these goals (see examples [19–21]). In this article, we examined the application of two types of spatial model selection techniques, Bayesian model selection based on information criteria (BMS) and Bayesian model averaging (BMA) [22, 23] to choose appropriate estimates in the small area COVID-19 transmission modeling. This can be achieved by assigning prior probability distributions to each of the possible parameters or models. For BMS, we then choose the parameter or model associated with the largest posterior information criteria while, in the BMA method, average posterior parameters or models are calculated based on the posterior model probabilities.

In this study we aimed to develop a generic and robust methodology for estimating spatiotemporal dynamic measures that can be instantaneously computed for each location and time within a Bayesian model selection and averaging framework. The proposed spatiotemporal reproduction number can also be linked to the effective reproduction number defined in [8, 24] as the weighted sum over the study units. The proposed methodology was described in the next section with a simulation study to demonstrate robustness of the method. A real-world case study was also provided of an application to COVID-19 national surveillance data in Thailand.

## Methods

### Temporal and spatiotemporal reproduction numbers

To evaluate the dynamic situation, it is crucial to accurately detect transmission changes and assess the impact of implemented interventions over time. There are two common ways to define the temporal measures in terms of reproduction number. The first quantity is the case reproduction number [25, 26] which is appropriate for retrospective surveillance data to understand how individuals infected at different time points contributed to the spread. This is a more natural choice for analyses that consider heterogeneity among individuals. For example, the case reproduction number can be adapted to incorporate data on observed transmission chains [25] or to produce age-structured estimates, given an age-structured contact matrix [27]. The instantaneous reproduction number is perhaps more suitable for estimating the reproduction number of the infected population on specific dates, particularly when the goal is to study how interventions or other extrinsic variables have an effect on the disease transmission at a given time. Conceptually, the case reproductive number may not be appropriate for timely estimation but might be useful in retrospective modeling, in particular for those involving individual risk factors.

More formally, the instantaneous or effective reproductive number, *R*_*t*_, is defined as the expected number of secondary infections occurring at time *t*, divided by the number of infected individuals, each scaled by their relative infectiousness at time *t* (an individual’s relative infectiousness is based on the generation interval and time since infection) [8, 26]. The generation time can be difficult to observe and a serial interval, the time from illness onset in the primary case to illness onset in the secondary case, is often adopted instead [18, 28]. The instantaneous reproduction number can be calculated using a published method [8, 26, 29] following the renewal equation in which the series of expected incidence arise from $Poisson\left( {R_{t} \sum\nolimits_{l}^{L} {y_{t - l} w_{l} } } \right)$ where *y*_t_ is the incidence at time *t*. From this, a data distribution given a set of model parameters can be calculated, as well as the posterior distribution of *R*_*t*_ given collected observations of incidence and knowledge of the serial intervals or weights,$\{ {\varvec{w}}_{l} \}$ where *L* is the maximum time of the generation interval. Conceptually, this estimator describes the ratio of the number of new infections on day *t* to the number of individuals who became infected *l* days in the past and who may now be shedding the infection.

To account for spatial heterogeneity at local scales, let *y*_*st*_ be the number of new COVID-19 cases at location *s* and time *t* and the disease transmission is presumably modeled with a Poisson process. With the cases usually reported at a discrete time interval such as daily, and assuming the transmissibility remains constant in the time interval (*t*, *t* + 1], the incidence at location *s* and time *t* then follows a Poisson distribution with mean $\mu_{st} = R_{st} \sum\nolimits_{l}^{L} {y_{st - l} w_{l} }$ where *R*_*st*_ is a spatial extension of the effective reproduction number, here named *spatiotemporal reproduction number*. To account for spatiotemporal variables and extra variation,$R_{st}$ can be linked to a linear predictor consisting of local variables such as environmental and demographic factors and space–time random effects as $\log (R_{st} ) = \alpha + {\varvec{X}}_{st} {\varvec{\beta}}_{st} + u_{s} + v_{s} + \lambda_{t} + \delta_{st}$. There is an extensive literature on space–time random effect modeling (see examples [30, 31]). To specify prior distributions, the correlated (*u*_*s*_) and uncorrelated (*v*_*s*_) spatial components commonly have an intrinsic conditional autoregressive model and zero mean Gaussian distribution respectively. For separate temporal random effect (*λ*_*t*_) and space–time interaction (*δ*_*st*_) terms in the linear predictor, the temporal effect can be described using an autoregressive prior distribution allowing for a type of nonparametric temporal effect, often with a random walk prior distribution with one-unit lag. For the interaction term, the prior structure is usually assumed to be distributed as a zero mean Gaussian distribution.

### Bayesian model selection and averaging infectious transmission dynamics

As mentioned above, the estimation of reproduction numbers is dependent on the choice of the infectiousness weight profile,$w_{l}$, which is an important ingredient to determine transmission dynamics in the renewal equation. In practice, the standard distribution for the generation time or serial interval weight can be considered as a discretized non-negative distribution. Gamma and Log-Normal are common choices in reproduction number estimation of infectious diseases including COVID-19 [8, 32, 33]. However, misspecification of the interval weight can lead to bias and the estimation is also sensitive to the parameters of the distribution, e.g. mean and variance. For example, if the mean is presumably too high, the computed reproduction number can be greater than one and vice versa. The reproduction number can be highly susceptible to the misspecification especially during the early period of transmission due to the limited data.

There are a number of ways to account for the uncertainty in the parametric specification. One option is to resample the parameters over a range of plausible values [8] while prior distributions also have been applied to quantify the estimation [34]. Selecting appropriate parameter values is one of the most important aspects of the disease transmission measure, and this can become very challenging when spatiotemporal structures are present in the data. Many methods have been proposed and explored to achieve these goals (see examples [19, 21, 23]). In this work, we discussed the application of two types of spatiotemporal model selection technique, model selection and model averaging, within the Bayesian framework, to account for uncertainty in parametric specification of reproduction number estimation.

### Bayesian model selection

Posterior measures have been proposed to assess the model selection. The “model” in general can be referred to as model specification or different values of parameters. Since we focused on generation time identification, the model here was referred to as combinations of associated parameters in the general time interval used in the reproduction number calculation. To perform model selection, one can simply choose the model with the best evaluation measure. Model assessment criteria are useful to measure how consistent the data are with a given specification. To evaluate choices for generation interval parameters, our assessment was based on five metrics. The first two were error rates, bias and root mean squared error (RMSE), and the other three were posterior Bayesian model selection measures.

The first error rate was bias, computed as the average difference between the simulated (true) mean and its estimate across the simulated datasets in each scenario. It is desirable for this measure to be near zero. To investigate the variance information of the estimates we then also examined RMSE, summation of the variance of an estimate plus the square of its bias. This metric was computed as the squared root of the average squared difference between the simulated mean and its estimate across the simulation replications. For posterior measures of model selection, the first method was the conditional predictive ordinate (CPO) [35]. This metric is a cross-validation criterion for model assessment that is computed for each observation as $CPO_{st} = P(y_{st} \user2{|y}_{ - st} )$. Hence, for each observation the conditional predictive ordinate is the posterior probability of observing that observation when the model is fit using all data for an observation at location *s* and time *t*. Large values indicate a better fit of the model to the data, while small values indicate a bad fit of the model. The conditional predictive ordinate measure for each model then can be summarized as $CPO = \sum\nolimits_{t} {\sum\nolimits_{s}^{{}} {CPO_{st} } }$ with bigger values indicating a better model fit.

In full Bayesian model comparison, the deviance information criterion (DIC) is a common metric used to evaluate the overall goodness of fit of models. For any sample primary parameter value *θ*^*g*^ for the conditional likelihood, the deviance is $D(\theta_{st}^{g} ) = - 2\log (P_{{\theta_{st} |y_{st} }} (y_{st} |\theta_{st}^{g} ))$ and $\overline{D }$ is the average deviance over the *g* posterior samplers. The effective number of parameters (*pD*) is estimated as $pD = \overline{D} - D(\overline{\theta }_{st} )$, and finally, $DIC = \overline{D} + pD$. An additional measure of the same type is the Watanabe-Akaike information criterion, also known as widely applicable Bayesian information criterion (WAIC), which makes use of the posterior predictive distribution, as described by Watanabe [36] and Gelman, Hwang, and Vehtari [37], such that $WAIC = - 2(lpd - pD_{WAIC} )$ where $lpd = \sum\nolimits_{s} {\sum\nolimits_{t}^{{}} {\log \left( {{{\sum\nolimits_{{}} {\sum\nolimits_{t}^{{}} {P(y_{st} |\overline{\theta }_{st} \user2{)}} } } \mathord{\left/ {\vphantom {{\sum\nolimits_{{}} {\sum\nolimits_{t}^{{}} {P(y_{st} |\overline{\theta }_{st} \user2{)}} } } {(S \times T)}}} \right. \kern-0pt} {(S \times T)}}} \right)} }$ and $pD_{WAIC}$ is the summation of the variance of log-likelihood. Small values of the information criteria indicate a better fit of the model.

### Bayesian model averaging

The Bayesian model selection presented above is appropriate when there is a single model standing out. However, if this is not the case, model averaging might be a more suitable alternative method that can produce an option that forms an estimate averaged over plausible alternatives weighted by the model probabilities. To perform Bayesian model averaging, this method averages over *j* = *1,…,J* models, *M*_*1*_,…,* M*_*J*_, to find the posterior estimates for the reproductive number. Then, to account for uncertainty over the possible models, the posterior estimate from model averaging follows

1 $$P(\theta_{st} |{\varvec{y}}_{st} ) = \sum\nolimits_{j}^{J} {P(\theta_{st} |{\varvec{y}}_{st} ,M_{j} )} P(M_{j} |{\varvec{y}}_{st} )$$

where $\theta_{st}$ is the parameter of interest,$P(M_{j} |{\varvec{y}}_{st} )$ is the model probability for model *j*, and $P(\theta_{st} |{\varvec{y}}_{st} ,M_{j} )$ is obtained by marginalizing the posterior distribution of the model parameters. By Bayes’ rule, the posterior selection probability for model *M*_*j*_ can be expressed as

2 $$P(M_{j} |{\varvec{y}}_{st} ) = \frac{{P({\varvec{y}}_{st} |M_{j} )P(M_{j} )}}{{\sum\nolimits_{j}^{J} {P({\varvec{y}}_{st} |M_{j} )P(M_{j} )} }}$$

where $$P({\varvec{y}}_{st} |M_{j} ) = \int {...\int {P({\varvec{y}}_{st} |{\varvec{\theta}}_{st} ,M_{j} )} } P({\varvec{\theta}}_{st} |M_{j} )d{\varvec{\theta}}_{st}$$.

With non-informative prior distribution on model averaging, one can assume a uniform prior probability across the model choices, i.e., $P(M) = P(M_{j} )\,\forall j$. The model probabilities can be estimated using the information criteria [38, 39] and the model probability can be defined based on the deviance information criterion as $P_{DIC} (M_{j} |{\varvec{y}}_{st} ) = \frac{{e^{{ - DIC(M_{j} )}} }}{{\sum\nolimits_{j}^{J} {e^{{ - DIC(M_{j} )}} } }}$. Similarly, we can also specify the model weights using WAIC as $P_{WAIC} (M_{j} |{\varvec{y}}_{st} ) = \frac{{e^{{ - WAIC(M_{j} )}} }}{{\sum\nolimits_{j}^{J} {e^{{ - WAIC(M_{j} )}} } }}$. Lastly, since CPO is related to the model goodness of fit, an alternative to define the model probabilities is $P_{CPO} (M_{j} |{\varvec{y}}_{st} ) = \frac{{CPO(M_{j} )}}{{\sum\nolimits_{j}^{J} {CPO(M_{j} )} }}$. In the next section, we conducted a simulation study with example data to demonstrate the performance of the proposed methodology with simulated ground truth and real national surveillance data.

## Results

### Simulation study

Thailand was one of the first countries outside China to be affected with COVID-19. It was successfully contained in Bangkok for the first few months. However, this was followed by cluster outbreaks in sport and entertainment events, and appearance of the disease in all provinces across the country. The proposed spatiotemporal reproduction number was developed as a surveillance tool to monitor disease dynamics at local scales described in the previous sections. In this part, a simulation study was conducted to assess our proposed methodology. The simulation data were generated without covariates in different situations with various space–time magnitudes of transmissibility. The district map of Bangkok, Thailand, was used as a basis for the simulation map to represent the disease transmission. This capital province has 50 districts (*s* = 1–50) with a reasonably regular spatial distribution. The simulated COVID-19 incidence was generated for 30 days (*t* = 1–30) in four different district groups with distinct levels of reproduction number.

**Fig. 2:**
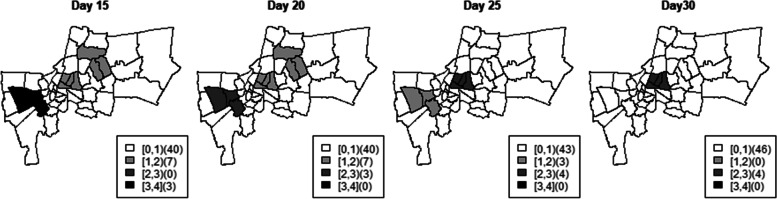
Maps of simulated *R*_*st*_

**Fig. 3:**
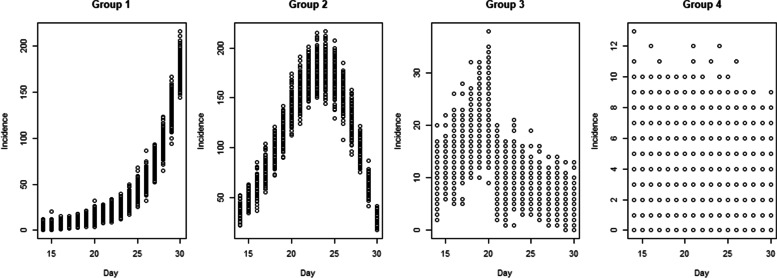
Simulated incidence in district groups with different disease transmission levels

Figure 2 displayed the maps showing locations of simulated $R_{st}$ of each district group on days 15, 20, 25 and 30. The simulated cases in each district group with different degrees of infection transmissibility was shown in Fig. 3 in which each dot represents a simulated incidence from a given simulation set. The first group (middle region in Fig. 2) was simulated with increasing levels of disease transmission as $R_{st} = 1 + (t \times 0.1)$. The *R*_*st*_ was assumed to grow each time period by size 0.1. Then simulated case counts with an exponential increase were generated in this scenario to represent regions with an outbreak (group 1, left panel in Fig. 3). The second district group (western region in Fig. 2) was assumed to have decreasing magnitudes simulated as $R_{st} = 4.0 - (t \times 0.2)$. As can be seen in Fig. 3 (group 2, second panel from the left), the incidence in this scenario increased at the beginning due to strongly positive force of infection but decreases afterwards. In the third scenario (eastern region in Fig. 2), *R*_*st*_ was assumed to be 1.8 until day 12, reducing to 0.6 thereafter. This scenario represented an effective intervention being introduced to control an outbreak. The rest of the districts were assumed to have a constant controlled infection rate at *R*_*st*_ = 0.9 over the time periods.

**Fig. 4:**
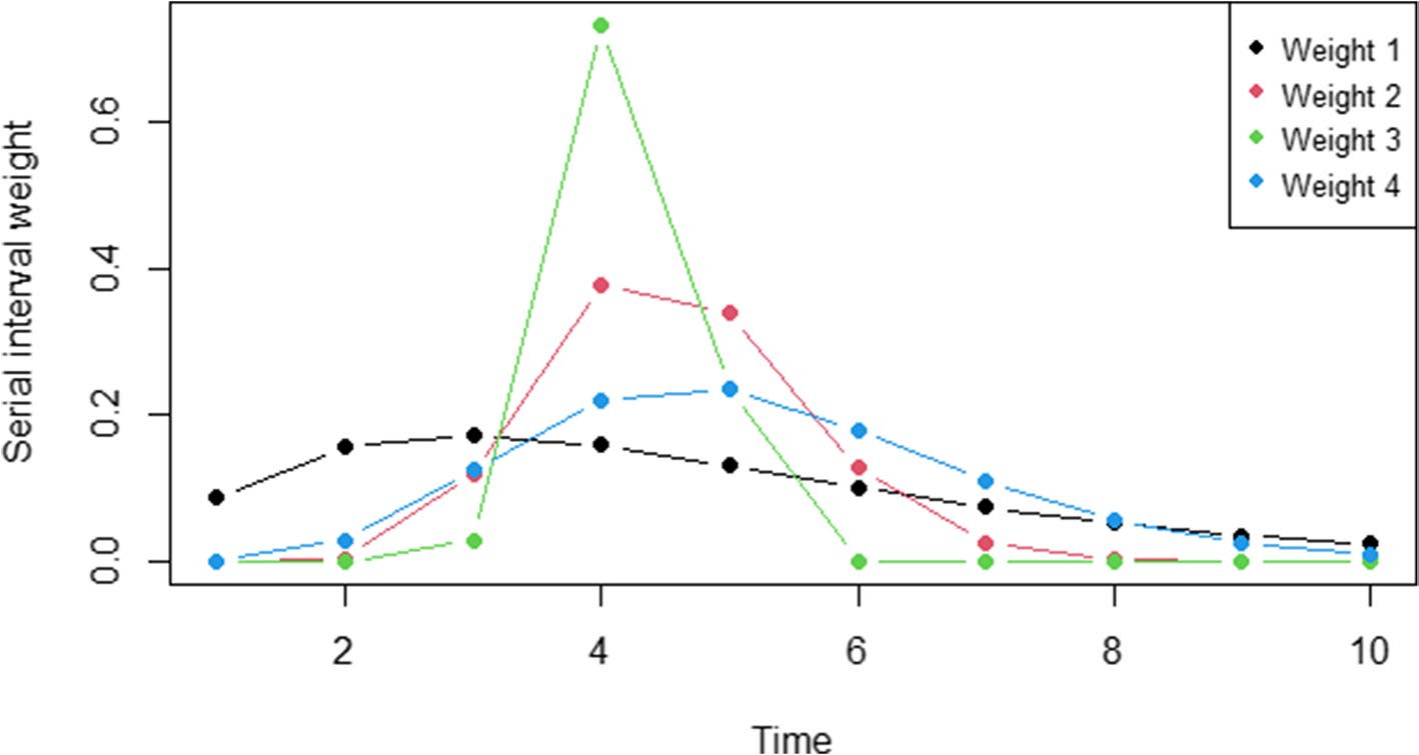
Plots of serial interval weights in the simulation study

The reproduction number calculation was dependent on the choice of serial intervals, which was an important ingredient in the renewal equation. Basing our simulation on the previous spatiotemporal study of COVID-19 transmission in Thailand [33], four serial interval weights (mean and standard deviation (SD)), closest to the overall mean of the basic reproduction number, were selected for this study. The four weights were weight 1 with mean = 4.7 and SD = 2.9; weight 2 with mean = 4.56, SD = 0.95; weight 3 with mean = 4.22, SD = 0.4; and weight 4 with mean = 5.2, SD = 1.72. For the discrete serial weights, $w_{l}$, were then drawn from a Gamma distribution with the parameter sets with the maximum infectious time, *L*, of 10 days. Figure 4 depicted the selected serial interval weights used in the simulation study.

One hundred simulated incidence datasets were generated with the number of newly infected people as 4 for the first 10 days. For days *t* > 10, the new cases $y_{it}$ were sampled from a Poisson distribution for each location with mean $\mu_{st} = R_{st} \sum\nolimits_{l = 1}^{10} {w_{sl} \mu_{st - l} }$. The prior distribution for precision parameters was set as a Log-Gamma (0.01, 0.01) distribution. In general, parameter estimates for this modeling framework can be computed from converged posterior samples using sampling-based algorithms such as Markov chain Monte Carlo (MCMC). However, since timeliness is an important feature of infectious disease surveillance, especially for emerging diseases, with the multi-dimensional model set up, MCMC makes high computational demands. A alternative approach to infer parameters in this context is the integrated nested Laplace approximation (INLA) [40]. With optimized numerical routines for performing the above computations, the proposed methodology was then implemented using the numerical Laplace approximation in the R-INLA package.

**Fig. 5:**
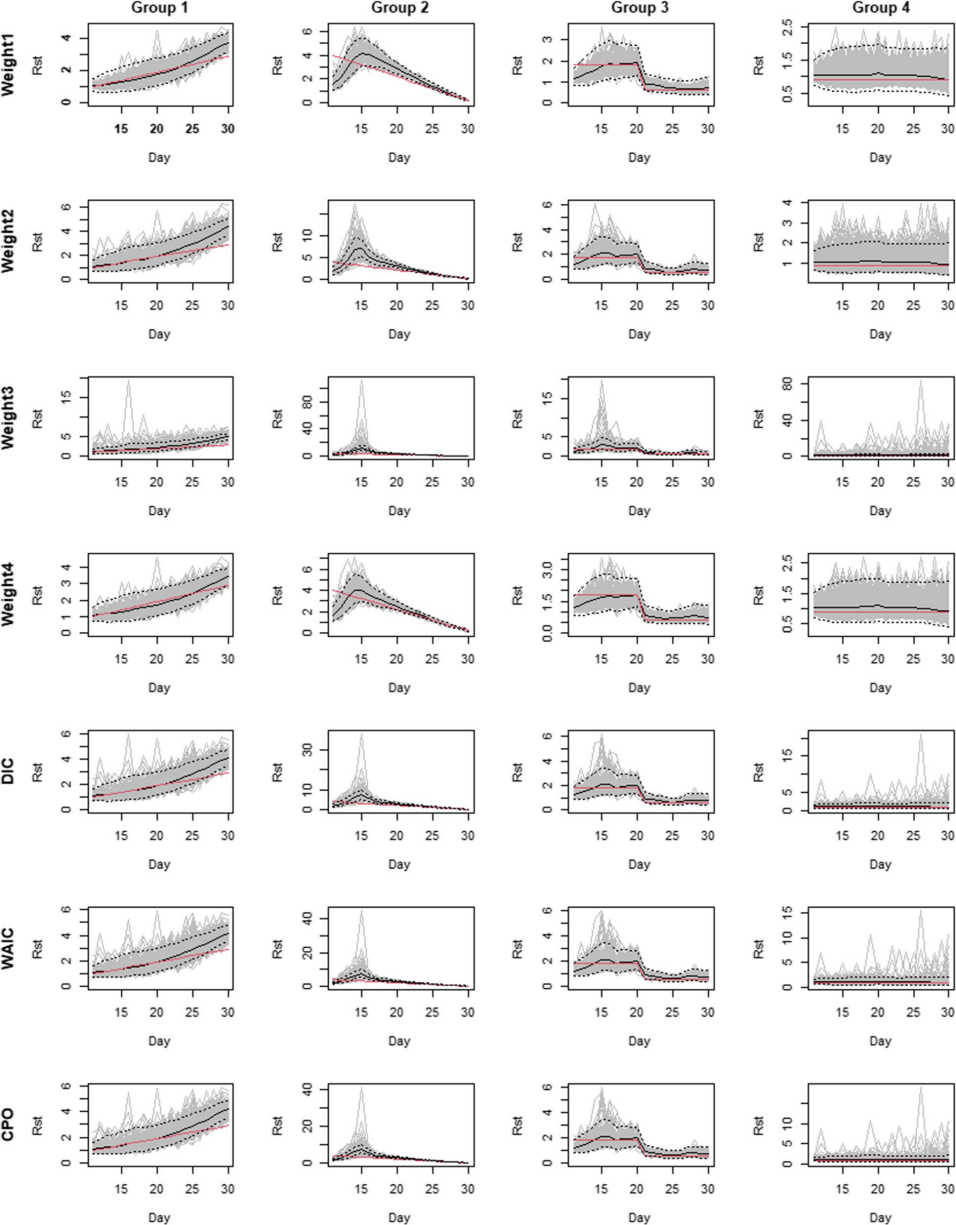
Plots of the posterior *R*_*st*_ estimates of district groups with different serial interval assumptions. The black lines show the estimated means with dashed lines showing the corresponding 95% credible intervals. The grey lines display posterior realizations and the red lines are the true *R*_*st*_ used for simulation

The simulated and corresponding estimated spatiotemporal reproduction numbers for each group of districts with different model selection and averaging criteria were depicted in Fig. 5 while numerical comparison based on evaluation metrics was shown in Table 1. Overall, the proposed method allowed for estimation of the constant reproduction number used in group 4 while the constant changes in $R_{st}$ were detected in both increasing (group 1) and decreasing (group 2) forces of infection. The method could also identify a rapid change in transmissibility, perhaps due to intense interventions such as lockdown policy (group 3). In terms of model selection, the serial interval weights 1 and 4 could best recover the simulated transmissibility, slightly better than weight 4, with the smallest bias and MSE and best goodness of fit criteria followed by weights 2 and 3 respectively. The model averaging yielded decent results between weight 3 and weights 1 and 4, similar to weight 2.

**Table 1:** Model comparison in the simulation study under evaluation metrics

Evaluation	Generation time weight				Model averaging		
Metric	Weight 1	Weight 2	Weight 3	Weight 4	DIC	WAIC	CPO
Bias	0.108	0.196	0.391	0.114	0.206	0.211	0.208
MSE	0.106	0.347	1.867	0.113	0.355	0.397	0.380
DIC	2249.569	2275.080	2287.286	2255.629	2275.299	-	-
WAIC	2223.215	2249.102	2260.929	2232.264	-	2251.752	-
CPO	63.675	61.797	60.647	63.670		-	62.532

**Fig. 6:**
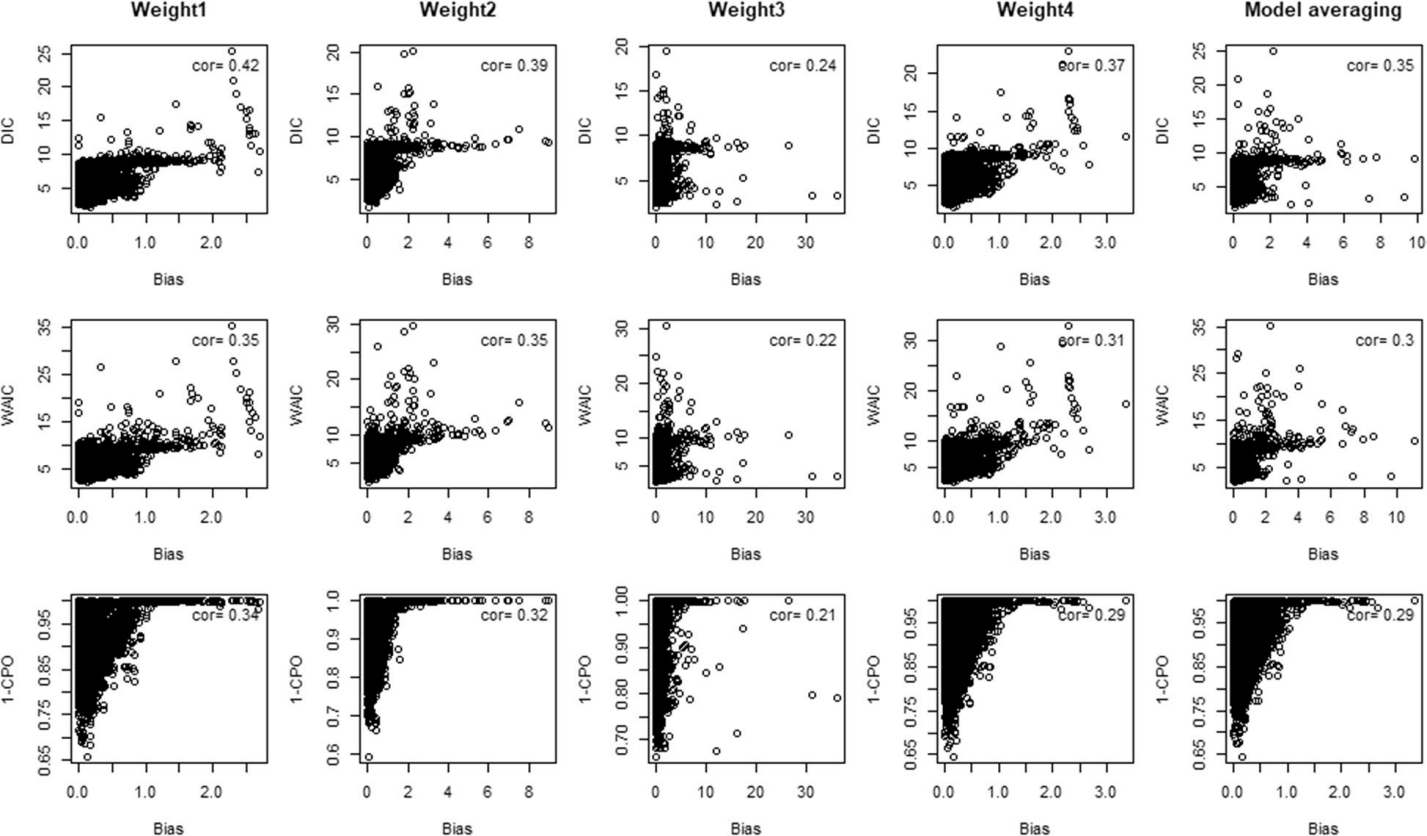
Plots of model selection and averaging criteria against absolute bias with correlation estimates of different serial interval weight assumptions

**Fig. 7:**
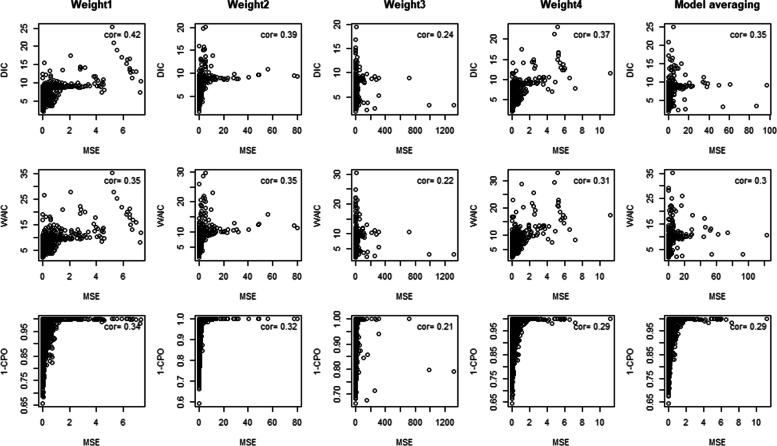
Plots of model selection and averaging criteria against MSE with correlation estimates of different serial interval weight assumptions

The estimation depends on the choice of infectiousness weight profile and this may not be feasible to assess using error rates in real surveillance situations. Figures 6 and 7 showed the correlation plot of model selection and averaging criteria against absolute bias and MSE with Spearman’s correlation estimates of different serial interval weight assumptions. All serial interval weights and averages based on model fit measures had positive correlation with estimation errors (upper right in Figs. 6 and 7). The correct serial interval, weight 1, had the best correlation across serial intervals and model selection criteria while DIC yielded the highest correlation with the estimation errors and might be useful as a selection measure for serial interval in practice.

### Case study of COVID-19 national surveillance

In Thailand, the COVID-19 infection firstly occurred in Bangkok in January 2020, most newly reported cases were related to transmission outbreaks including those who had returned from overseas or had been exposed to many people linked to tourism businesses. Most cases were middle-aged males because so many cases were related to boxing stadia, entertainment venues and to attendance at religious events [41]. COVID-19 was then successfully under controlled in Bangkok for the first two months. However, it was followed by disease clusters in sport and entertainment events, and occurrences of the infection in all provinces across the country.

Disease transmissibility can differ across places since the contact patterns among individuals vary due to differences in the local factors (e.g. weather and population density) and human behavior (e.g. personal protection, working pattern and travel). Thus, the spatial variation of disease transmission between locations should be integrated to provide more granular information for policy makers in order to effectively monitor high-risk areas. This is potentially helpful for prioritizing medical and public health resources, especially during disease outbreaks. To demonstrate the developed method in practice, the data in this case study were from confirmed provincial COVID-19 cases in Thailand from January 12^th^ 2020 through July 31^st^ 2020 provided in the daily reports of the Department of Disease Control, Thai Ministry of Public Health. Suspected cases with COVID-19 infection were identified in designated health facilities and confirmed at certified laboratories by virus polymerase chain reaction of nose and throat swabs. The place of diagnosis and demographic data were obtained from the official website of the Digital Government Development Agency (https://data.go.th/dataset/covid-19-daily). The data used in this case study are publicly available and ethical approval was not required.

The developed spatiotemporal reproduction number was applied to the Thai national surveillance data. Table 2 showed the comparison of model selection and averaging of serial interval weights under different evaluation metrics using the national COVID-19 surveillance data. The serial weight 1 performed best based on model selection and averaging criteria, which is consistent with findings in previously estimated basic reproduction numbers for the country [33]. To further estimate the temporal or effective reproduction number within the proposed framework, a temporal reproduction number could also be derived from the proposed spatiotemporal reproduction number which could be defined as the weighted sum of *R*_*st*_ over the study units as $R_{t} = \frac{{\sum\nolimits_{s} {\left( {R_{st} \times \left( {\sum\nolimits_{l} {y_{st - l} w_{l} } } \right)} \right)} }}{{\sum\nolimits_{s} {\sum\nolimits_{l} {y_{st - l} w_{l} } } }} = \frac{{\sum\nolimits_{s} {y_{st} } }}{{\sum\nolimits_{s} {\sum\nolimits_{l} {y_{st - l} w_{l} } } }} = \frac{{y_{t} }}{{\sum\nolimits_{l} {\sum\nolimits_{s} {y_{st - l} w_{l} } } }} = \frac{{y_{t} }}{{\sum\nolimits_{l} {w_{l} y_{t - l} } }}$. This calculation also yielded a similar form of the effective reproduction number defined in [8, 24].

**Table 2:** Model selection and averaging comparison under different evaluation metrics using the national COVID-19 surveillance data

Evaluation	Generation time weight				Model averaging		
Metric	Weight1	Weight2	Weight3	Weight4	DIC	WAIC	CPO
DIC	878.252	892.903	899.664	881.804	894.425	-	-
WAIC	874.034	891.296	899.694	878.431	-	895.822	-
CPO	477.727	408.128	356.711	466.111	-	-	467.593

**Fig. 8:**
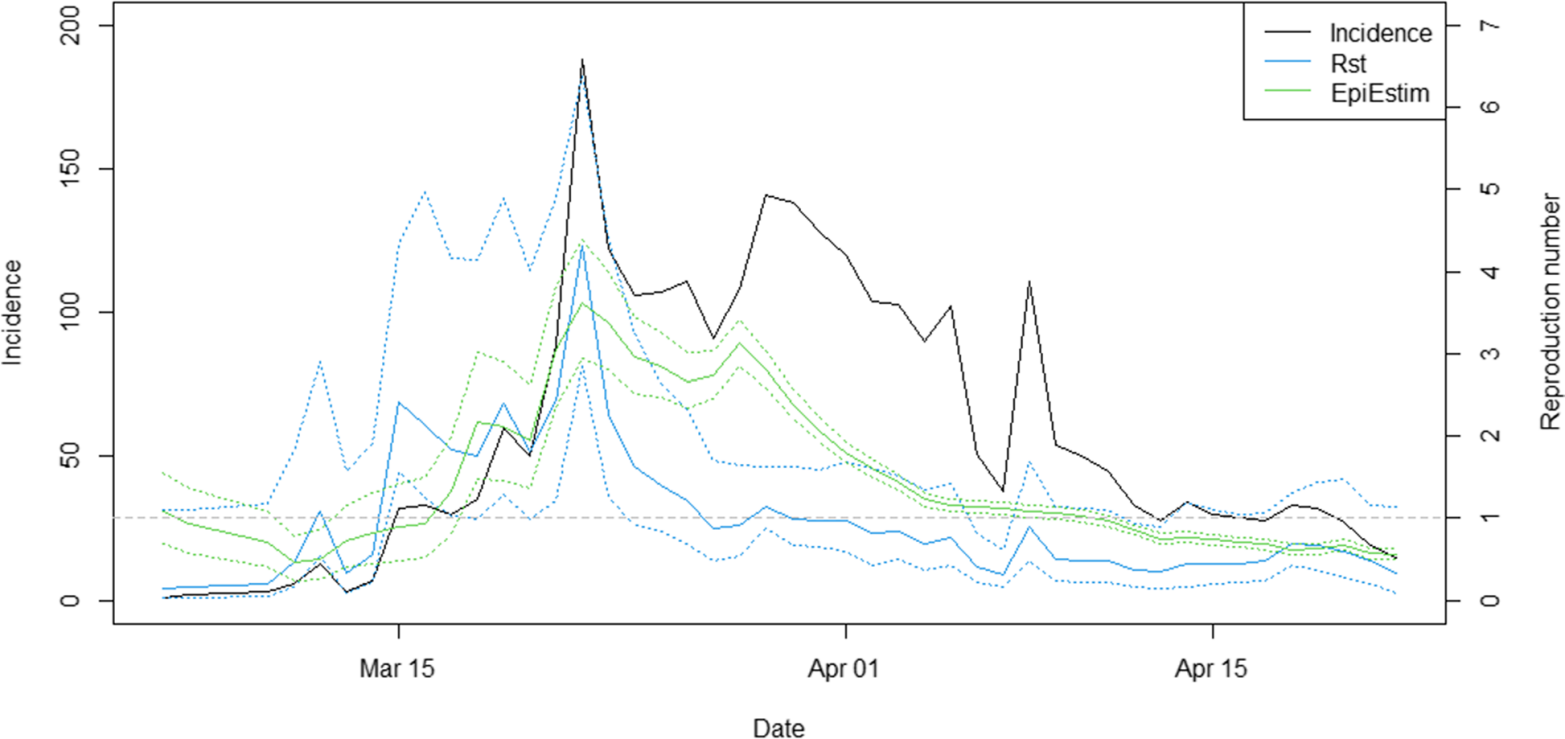
Plots of COVID-19 incidence at country level (black), temporal reproduction numbers calculated from *R*_st_ (blue) and EpiEstim (green) with 95% credible intervals (dash)

Figure 8 showed the number of new cases for the whole country (black) with an estimated *R*_*t*_ from the proposed model (blue) and the EpiEstim software (green) [8, 42] over March–April 2020. After the boxing stadium and entertainment venue events presumably acted as outbreak spreaders, the numbers of new cases increased until mid-March. Then the number of new cases sharply increased after about one week with a large jump in *R*_*t*_. The estimates of *R*_*t*_ using both methods had similar trends as depicted in Fig. 8 suggesting that the outbreak during Mid-March was controlled by strict public health policies represented by decreasing* R*_*t*_ towards the middle of April where estimated *R*_*t*_ < 1. The number of new cases then continued fluctuating thereafter likely due to imported cases returning from overseas. This could be partly related to testing capacity and infection residuals. However, the Thai government had also implemented travel restrictions including permission to enter or transit through Thailand since May. Though the temporal reproduction numbers estimated from both methods had a similar overall behavior, the *R*_*t*_ from the proposed model seemed to drop after the first wave while the EpiEstim estimate appeared to have a lagged elevated pattern. In addition, the *R*_*t*_ from the proposed model yielded a wider credible interval. This might be due to the variation in spatiotemporal random effects included in the *R*_*st*_ which didn’t account for in EpiEstim.

**Fig. 9:**
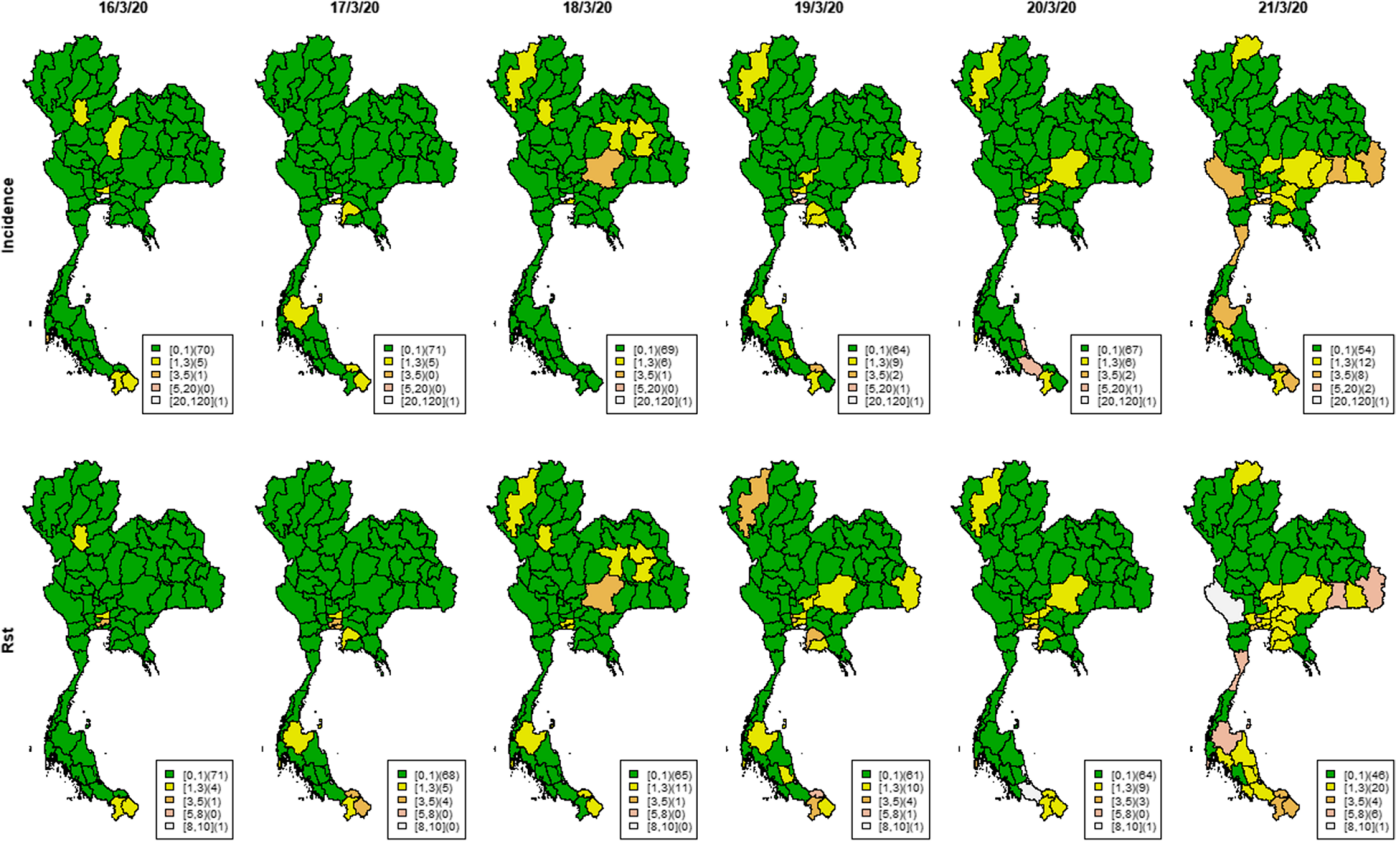
Maps of Thai provincial COVID-19 incidence and *R*_*st*_ during March 16^th^ – March 21.^st^ 2020

**Fig. 10:**
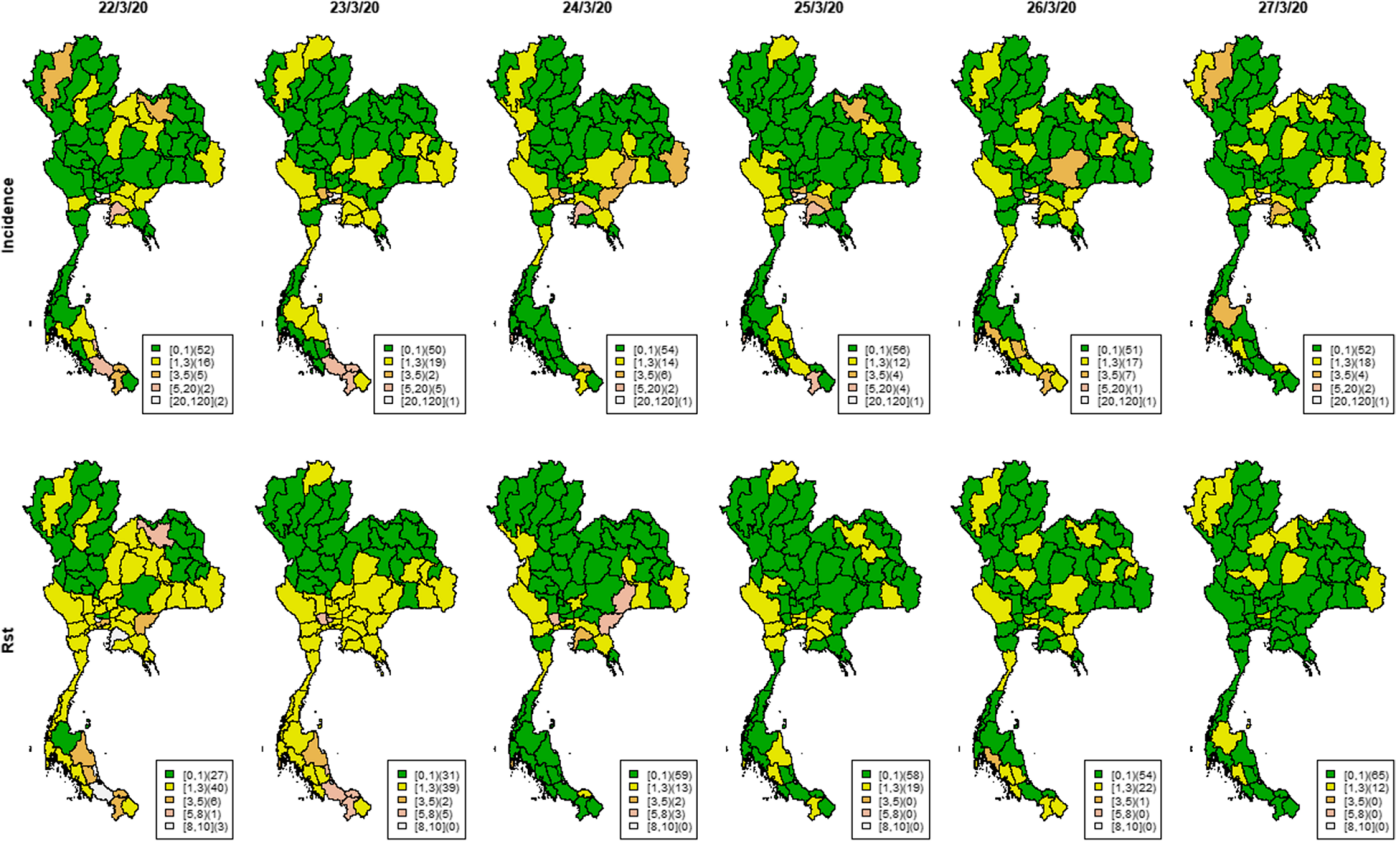
Maps of Thai provincial COVID-19 incidence and *R*_*st*_ during March 22^nd^ – March 27.^st^ 2020

The countrywide spread was also reflected in the incidence and *R*_*st*_ maps in Figs. 9 and 10. Many provinces had few or no cases on March 16^th^. Then there were more cases a few days later on March 20^th^, increasing further on March 21^st^ with high *R*_*st*_ in several provinces. The incidence then started to decrease after March 23^rd^. During the first big outbreak from March 16^th^ – 27^th^ 2020, there was increased *R*_*st*_ in the south and west of the country, and some new disease clusters spreading over the central region and along the Thai-Cambodia border. This contrasted with the isolated hot spots also found in Northern areas. As demonstrated, this proposed methodology might be helpful in real time surveillance of infectious diseases to identify local transmission requiring more immediate attention to prevent wider spread.

## Discussion

When a new infectious disease epidemic emerges, a crucial challenge for disease control preparation is that the situation may be very dynamic. Health authorities need to make decisions based on limited evidence. The novel coronavirus infection has been the primary public health concern in Thailand since early 2020 with the declaration by WHO of a global health emergency. Due to the lack of effective treatment and sufficient vaccines, disease surveillance has been a major public health intervention for COVID-19. The reproduction number is a key threshold widely used to assess the transmission dynamics of an emerging infection, however the basic form of reproduction number, *R*_*0*_, yields the average number of secondary cases generated per case in a fully susceptible population likely to be in the early phase of epidemics when interventions and behavior changes have not affected the transmission dynamics.

For real time surveillance aiming to rapidly quantify disease transmissibility over time and assess the impact of policy implementation or other extrinsic factors on transmission, the instantaneous or effective reproduction number is currently the most suitable tool [18]. However, this concept assumes that the population is geographically well mixed which may not be useful for real time surveillance at fine spatial scales. To account for heterogeneity of transmission intensity, we thus extended the concept and developed the spatiotemporal reproduction number which requires minimal parametric assumptions about the underlying disease transmission process and is practically appropriate for real time infectious disease outbreak detection.

Another issue when estimating reproduction numbers from observed data is misspecification of the generation interval which can be a large potential contributor to estimation bias. Although the intrinsic generation interval is required to correctly define the relationship between *R*_*t*_ and disease incidence, the generation interval is difficult to measure and the reproduction numbers are often approximated from serial intervals. To accommodate for the parametric specification [43], in this study we then applied and compared Bayesian model selection and averaging methods to address the issue using national surveillance data. When there are several candidates, one can select the most suitable conditions based on model fitting criteria or choose to average over candidate models. However, the essential selection procedure is not available in widely used software packages. In addition, those methods are usually used for temporal reproduction number estimation whereas our methodology was developed for disease dynamic estimation in both space and time dimensions.

Based on the simulation results with qualitative assessment, we believe that the proposed model selection technique is an important tool to help accurately estimate disease dynamics and outperformed model averaging in terms of specifying the appropriate serial interval parameters. The model selection chose the true serial interval when included in our study, however the second best would otherwise be selected. On the other hand, model averaging yielded the weighted outcome which could be less accurate than model selection. Nonetheless, model averaging might be appropriate if it was believed there were more than one best suitable condition. To generalize this concept to real surveillance situations, an aim of parametric specification was to select the appropriate condition with least error rates in estimation which we would not be known in practice. The mean absolute and square error rates were compared and correlated against model selection criteria which could be computed with observed data. The selection criteria had positive correlation with the error rates while DIC and WAIC had similar results and yielded the highest correlation. Hence, the information criteria might be useful when choosing the appropriate parameters in practice for real surveillance activities. This also had demonstrated and been consistent with the case study of Thai national surveillance data [33].

Precise estimation of reproduction numbers needs complete epidemiological data over time. However, lags in case reporting can happen in the notification system which is a result of a chain of events from infection until report at the local, regional or national public health services. Accounting for the lag in a surveillance system, which can be spatially varying, is therefore key for disease control planning as incomplete and delayed information can undermine efforts to deliver early warnings and real time detection required for an effective response to the public health threat. A simple approach is to shift the observed time by the mean delay. Nonetheless, this approach would work well only if the delay to observation is not highly variable and the mean delay is known. In addition, the shifting by a fixed amount of time does not account for uncertainty or individual variation in delay times. To address the delay issue in real time surveillance, more complex methods correcting for delays in report time series such as deconvolution or nowcasting [18, 44] can be added to the developed methodology which can potentially improve estimation accuracy.

Though the proposed method demonstrated robust performance in both the simulation and case study, it should be noted that the nature of emerging infections presents a lot of clinical and epidemiological complexity. There is a need for further studies, for example, on persistence of virus circulation and on ecological factors, including characterizing immunological cross-reaction, which could shorten or prolong the infection. Across both clinical and epidemiological studies, it is also important to evaluate the effects of host, viral, and population-health relationships for fuller understanding of the disease mechanism. However, the proposed methodology can serve as a flexible platform to incorporate those available potential clinical and epidemiological determinants that drive the disease risk.

## Conclusions

New emerging diseases are public health crises in which policy makers have had to make decisions in the presence of massive uncertainty. As presented, the proposed methodology extended the concept of effective reproduction number to disease surveillance at finer scales to account for spatial heterogeneity of disease transmission. The method yielded robust estimation in several simulated scenarios of force of transmission with computing flexibility and practical benefits. Thus, this development can be suitable and useful for surveillance applications especially for newly emerging diseases. Nonetheless, we also believe that ongoing modelling and monitoring efforts should remain to continuously evaluate public health interventions. New emerging or re-emerging disease outbreak clusters have happened across the globe. As this pandemic continues to develop and the risk changes on both local and global scales, hopefully our work can provide an addition to the greater picture for surveillance activities and facilitate policymaking for disease control at the individual and population levels.

## Supplementary Information

**Additional file 1.**

## Data Availability

The datasets analyzed during the current study are available in the official website developed by the Digital Government Development Agency (https://data.go.th/dataset/covid-19-daily).

## Abbreviations

ICAR: Intrinsic Conditional Autoregressive model
*R*_*t*_: Temporal or effective reproduction number
*R*_*st*_: Spatiotemporal reproduction number
MSE: Mean Squared Error
DIC: Deviance Information Criterion
MCMC: Markov chain Monte Carlo
RMSE: Root Mean Squared Error
CPO: Conditional predictive ordinate
