# Supplementary material for: Spatiotemporal reproduction number with Bayesian model selection for evaluation of emerging infectious disease transmissibility: an application to COVID-19 national surveillance data
Source: BMC Med Res Methodol. 2023 Mar 14;23:62. doi: 10.1186/s12874-023-01870-3 (PMC10010957; doi:10.1186/s12874-023-01870-3)
Supplement: Supplementary file 1 — Additional file 1. [file 12874_2023_1870_MOESM1_ESM.docx]

**Supplementary document**

**Spatiotemporal reproduction number with Bayesian model selection for evaluation of emerging infectious disease transmissibility: An application to COVID-19 national surveillance data**

**S1 R code to run for the simulation study**

L <- 10 #maximum serial interval length

t2 <- L+1 # >= L

t1 <- t2-(L-1)

for(k in 1:n.sim){ # set up the number of generated data sets

for(t2 in (L+1):T){

t1 <- t2-(L-1)

Rs <- matrix(rep(0,I*t2,),nrow=I)

Rs.l <- matrix(rep(0,I*t2),nrow=I)

Rs.u <- matrix(rep(0,I*t2),nrow=I)

for(m in 1:4){

y <- as.vector(y.sim[,t1:t2,k])#by column first

E <- rep(as.vector(w[,m]%*%t(y.sim[,t1:t2,k]+0.01)),L)

ID.time <- numeric(0)

for(i in 1:L){

ID.time<- append(ID.time,rep(i,I))

}

ID.area <- rep(seq(1,I),L)

ID.area.time <- seq(1,I*L)

data <- data.frame(y=y, E=E, ID.area=ID.area, ID.time = ID.time, ID.area.time=ID.area.time)

formula<- y ~ 1 + f(ID.area,model="bym", graph=dis.bkk.adj,

hyper=list(prec.unstruct =list(prior="loggamma",param=c(0.01,0.01)),prec.spatial = list(prior="loggamma",param=c(0.01,0.01)))) +

f(ID.time,model="rw1",hyper = list(prec = list(prior="loggamma",param=c(0.01,0.01)))) +

f(ID.area.time,model="iid",hyper = list(prec = list(prior="loggamma",param=c(0.01,0.01))))

mod1 <- inla(formula,family="poisson",data=data,E=E,control.predictor=list(compute=TRUE),control.compute=list(waic=TRUE,dic=TRUE,cpo=TRUE))

##measures##

Rsim[,t2,m,k,1] = matrix(exp(mod1$summary.linear.predictor[,1]),nrow=I,ncol=L)[,L]

Rsimu[,t2,m,k,1] = matrix(exp(mod1$summary.linear.predictor[,5]),nrow=I,ncol=L)[,L]

Rsiml[,t2,m,k,1] = matrix(exp(mod1$summary.linear.predictor[,3]),nrow=I,ncol=L)[,L]

l.dic[,t2,m,k,1] = matrix(mod1$dic$local.dic,nrow=I,ncol=L)[,L]

l.waic[,t2,m,k,1] = matrix(mod1$waic$local.waic,nrow=I,ncol=L)[,L]

l.cpo[,t2,m,k,1] = matrix(mod1$cpo$cpo,nrow=I,ncol=L)[,L]

al.dic[,t2,m,k,1] = apply(matrix(mod1$dic$local.dic,nrow=I,ncol=L),1,sum)

al.waic[,t2,m,k,1] = apply(matrix(mod1$waic$local.waic,nrow=I,ncol=L),1,sum)

al.cpo[,t2,m,k,1] = apply(matrix(mod1$cpo$cpo,nrow=I,ncol=L),1,sum)

dic.sim[t2,m,k,1] = mod1$dic$dic

waic.sim[t2,m,k,1] = mod1$waic$waic

cpo.sim[t2,m,k,1] = sum(mod1$cpo$cpo)

mlik.sim[t2,m,k,1] = mod1$mlik[1]

}
